# Interdependent Utilities: How Social Ranking Affects Choice Behavior

**DOI:** 10.1371/journal.pone.0003477

**Published:** 2008-10-22

**Authors:** Nadège Bault, Giorgio Coricelli, Aldo Rustichini

**Affiliations:** 1 Institut des Sciences Cognitives, Centre de Neuroscience Cognitive, CNRS UMR5229, Université Lyon1, Bron, France; 2 Center for Mind/Brain Sciences, CIMeC, University of Trento, Mattarello, Trento, Italy; 3 Department of Economics, University of Minnesota, Minneapolis, Minnesota, United States of America; James Cook University, Australia

## Abstract

Organization in hierarchical dominance structures is prevalent in animal societies, so a strong preference for higher positions in social ranking is likely to be an important motivation of human social and economic behavior. This preference is also likely to influence the way in which we evaluate our outcome and the outcome of others, and finally the way we choose. In our experiment participants choose among lotteries with different levels of risk, and can observe the choice that others have made. Results show that the relative weight of gains and losses is the opposite in the private and social domain. For private outcomes, experience and anticipation of losses loom larger than gains, whereas in the social domain, gains loom larger than losses, as indexed by subjective emotional evaluations and physiological responses. We propose a theoretical model (interdependent utilities), predicting the implication of this effect for choice behavior. The relatively larger weight assigned to social gains strongly affects choices, inducing complementary behavior: faced with a weaker competitor, participants adopt a more risky and dominant behavior.

## Introduction

In the postal code lottery conducted in the Netherlands your ticket is linked to your postal code [1]. The lottery is very popular and this is perhaps due to the strong regret you would feel if you had not bought the ticket and your code was selected. However, a second strong emotion may be operating: if your code is selected, your neighbors who had bought the ticket will win the lottery, and envy would be added to regret.

We study here the link between emotions in individual and social settings. Recent research on the neural basis of regret [2], [3] suggests that this emotion has an important role in learning to evaluate our actions: the counterfactual thinking [4] (“I would have been better off by choosing the other option”) keeps a record for future use of the outcome of our past choices compared to the available alternatives. Envy may have a similar role, operating as the social analogue of regret (“I would have been better off by choosing the option he chose”). The issue we address is whether there is more to envy than this counterfactual thought. With envy we also keep track of our social status by coding the loss of social rank produced by an inferior outcome. This suggests an additional component of emotions associated with different relative outcomes, such as envy (for unfavorable differences, social losses) and gloating (for favorable ones, social gains)[5], [6]. We consider envy and gloating as complex, events-based emotions related to the fortune of others [5]. While envy refers to a comparison between someone's negative situation and another individual's positive situation, gloating refers to a comparison between someone's positive situation and another individual's less fortunate situation [6]. Thus envy and gloating both involve social comparison.

A rich research tradition in sociology [7], social psychology [8]–[12] and economics [13]–[15] has also demonstrated how concern for status strongly motivates human behavior. For example a major determinant of workers' effort is how their income is ranked within their firm [16]. More generally, happiness and well-being are strongly affected by the comparison between the individual's own income and the income of others [17]–[20].

From the Social Comparison Theory [9] we derive the insight that individuals use the comparison with others to evaluate their own opinion and abilities, or in other words that comparison is informative for the subject who makes the comparison. Hence individuals have an incentive to gather and process this information. Upward comparisons are motivated by self improvement (improving one's own abilities) which aims at enhancing social status [21], while the opportunity to compare with a less fortunate other enhances subjective well-being [22], [23].

Social actions, such as consumption, are used to communicate to others a signal about some private information that is relevant for the social ranking of the individual. The social signal has to be costly, or else it could be easily mimicked. For instance, according to Veblen [7], conspicuous consumption has to be wasteful. In our experiment, the cost is the distortion away from the choice that would maximize expected utility. This is induced by the concern for the public signal introduces.

The goal of this study was first to directly compare how individuals evaluate the outcome of their decision in private versus social contexts, with the hypothesis that for a given outcome, social context will enhance emotional responses due to social comparison. Second, the study was designed to investigate whether social and private emotions influence monetary decisions in different ways.

We theoretically (*Theory of interdependent utilities*, see **Methods** and **Supporting Text S1**) and experimentally disentangle the two components of these social emotions: (i) learning to evaluate our actions and (ii) keeping track of our ranking. The learning component is common to existing theories of regret [24]–[27]. In these theories, learning adjusts the probability of choosing an action depending on the difference between the total rewards that could have been obtained with the choice of that action and the realized total rewards. The theory of interdependent utilities that we adopt is a general form of the regret theory. When the foregone action is an action that was available but not chosen, the function gamma (see **Methods** and **Supporting Text S1**) represents regret/relief; when it is an action chosen by others, it represents envy/gloating. The emotions we study may be considered as the affective evaluation of a difference between an expected and a realized value. This general hypothesis assigns to emotions a functional role, similar to the one fulfilled by the adjustment to prediction error: in this view, learning is adaptive learning, and it adjusts the current evaluation of an action to a new and updated value. Emotions keep track of the difference between expected and realized value, and increase or decrease the value depending on the difference. Within this hypothesis, emotions do not necessarily interfere with rational decision making, and on the contrary they may implement it: they are a way of evaluating past outcomes to adjust choices in the future.

We show experimentally that the ranking component adds to the learning one, so that the social emotions have stronger effects than their private counterparts, they operate differently, and they affect our behavior in a deeper way.

**Figure 1:**
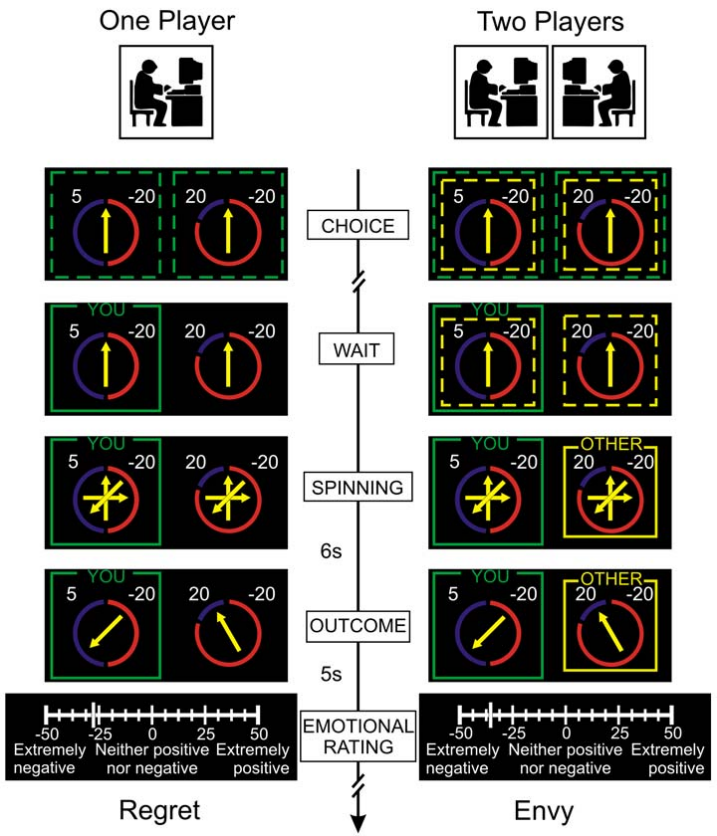
Experimental Design. Time line in the typical single (on the left) and two player trial. Numbers indicated outcomes, and the probabilities were represented by colored sectors of a circle. Each lottery was surrounded with one dotted square in the case of a one player trial or two dotted squares of different colors in the case of a two player trial. In one player trials, after they made their choice the square surrounding the chosen lottery became continuous and the other dotted square disappeared. In two player trials, the selected lottery was marked with a green square, and the lottery chosen by the other player (possibly the same) with a yellow square. The other player's choice was displayed after the participant's choice. After both players had made their choice, the participant observed an arrow spinning on both lotteries. Approximately 6 s later, outcomes were indicated by the final position of the arrows, showing how much he won and how much he would have won, and (on two player trials) how much the other won. Subjective emotional evaluation. At the end of each trial the participant indicated his subjective feeling on a scale from −50 (“Extremely Negative”), through 0 (“Neither Positive nor Negative”) up to +50 (“Extremely Positive”).

In our experiment participants choose among lotteries, with different levels of risk, and observe the choice that others have made. They are then informed of the monetary outcome of their choice and the choice of others, and have the opportunity in this way to experience regret and envy, or their positive counterparts (relief and gloating). Two players participated in each experimental session (**Fig. 1**). In each trial participants were first informed about the condition in which they were going to play, which could be one or two players. They were then presented with two lotteries and they had to choose one of the two. In the one player trial, the participant was then informed of the outcome of the lottery he had chosen and the outcome of the other lottery. In the two player trials after his choice he was informed of the choice of the other participant, which could be the same that he had selected or not. He was then informed of the outcome of the two lotteries: so he would be able to compare his outcome and the outcome of the other. After each trial, the player rated his subjective feeling on the outcome he had just observed, using a slider scale from extremely negative to extremely positive. Emotional arousal [28] was assessed by recording the skin conductance responses (SCR) and heart rate of all participants during the entire experiment. Physiological measures provide a robustness check for the subjective ratings.

Six events are possible on each trial, depending on the condition (one or two players), both players' choices (same or different) and on the outcome (gain or loss relative to the other lottery's outcome). In our classification, each event is associated with an emotion. We did not directly assess whether these discrete emotions were evoked in participants, thus the emotion terms are intended to label emotional events consistent with the context in which they occur, according to the appraisal theory of emotions [29]. Relief and regret are the events occurring in the one player trials when the payoff of the participant was larger or smaller than the one of the non chosen lottery; shared relief and shared regret occur in two players trials when both players made the same choice; and gloating and envy occur when their choices were different.

## Results

### Social competitive emotions are stronger than their private counterparts

**Figure 2:**
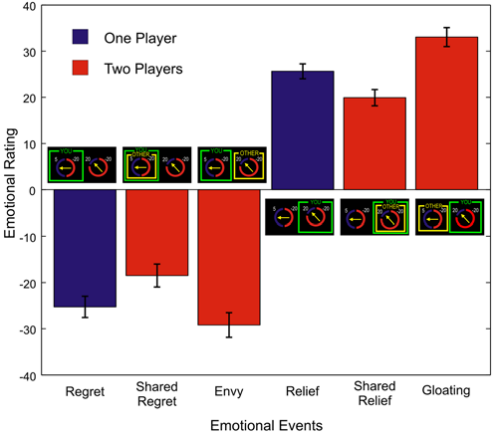
Emotional responses: Average subjective emotional evaluations for different events. The bars represent the average value (±SEM) of the subjective emotional evaluation given by participants in the different events. The pictures around the horizontal axis show the typical screen display seen by participants in the different events.

**Figure 3:**
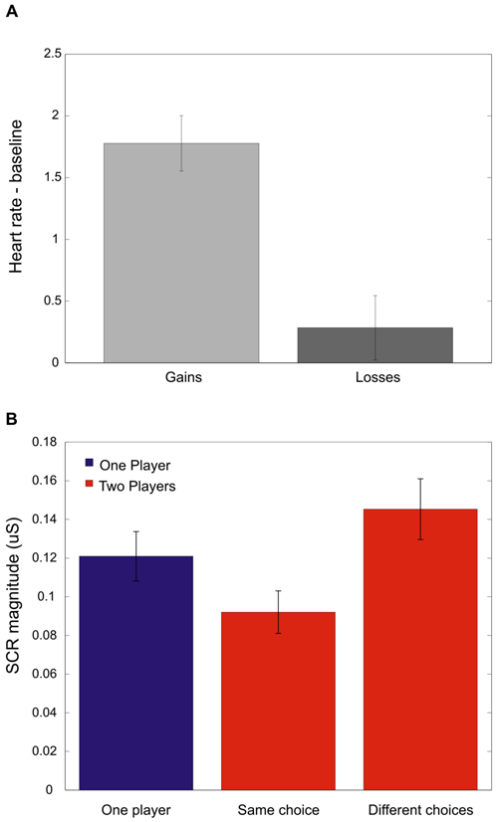
Physiological responses. (a) Variation in Heart Rate for positive and negative events. Vertical bars represent the average value (±SEM) of the participants' heart rate variation from baseline (2 seconds before the outcome), in beats per minute. (b) Magnitude of the skin conductance responses (SCR). The bars represent the SCR magnitude in microsiemens after the outcomes of the lotteries were displayed. Data are classified by condition for each participant: individual condition (regret and relief), social condition when both players made the same choice (shared regret and shared relief) and social condition when the payers chose different lotteries (envy and gloating). Table S1 reports the SCR magnitude for each emotional event.

The results of self evaluation of emotional state about the choice's outcome showed that relief, shared relief and gloating received an average positive score, while the other three events had a negative rating (**Fig. 2**, and **Table S1**). Different physiological responses corresponded to positive and negative emotional events; for instance, participants' heart rates were significantly higher for the three positive emotions compared with the negative ones (Wilcoxon Signed Rank Test (WSRT), *Z* = 4.283, *P* = 0.0001, **Fig. 3A**). SCR measures did not distinguish between positive and negative emotional events. A Friedman tests revealed a significant effect of the social context (with three levels, private, social same and different choices) for both positive (*χ^2^* = 30.33, *P*<0.001) and negative emotions (*χ^2^* = 29.19, *P*<0.001). Those in the two players condition received a stronger rating (larger in absolute value) than their correspondent in the single player trial. Specifically, gloating was stronger than relief (WSRT, *Z* = 4.03, *P*<0.001), and envy was stronger than regret (WSRT, *Z* = 2.75, *P* = 0.035). On the other hand, the shared emotions in the two player trials had a weaker rating than did their single player correspondent: relief was stronger than shared relief (WSRT, *Z* = 4.62, *P*<0.001), and regret was stronger than shared regret (WSRT, *Z* = 4.12, *P*<0.001, see **Tables S2** and **S3** for all tests). Even when controlling for the obtained outcome, the effect of social context still holds for both negative (*F*(3, 119) = 219.551, *P*<0.001) and positive emotions (*F*(3, 116) = 104.996, *P*<0.001). Notably, we did not find any gender differences, neither in the evaluation of the emotional events (P>0.4 for all 6 events), nor in total earnings (P>0.2) nor in choice time (P>0.4). The unobtained outcome of the chosen lottery might also modulate the subjective evaluation of the obtained outcome and result in the feeling of disappointment and elation [2]. However, emotional ratings are dominated by the comparison between the outcomes of the two lotteries. Moreover, the amplification effect in the envy and gloating events still operates even when controlling for the effect of the unobtained outcome of the chosen lottery (**Table S4**). To measure the arousal associated with the outcome evaluation in the different events we recorded participants' skin conductance responses (SCR). This auxiliary measure offers a strong support to our interpretation of the self-reported emotional evaluations. Data on self reporting rates and physiological responses are extremely consistent in our experiment. The correlation between the subjective rating and the SCR is high (r = 0.932) and significant (*P* = 0.0067). A Friedman test revealed a significant effect of the social factor (χ^2^ = 10.22, *P* = 0.005) on SCR measurements.

Comparisons between obtained and unobtained outcomes in the two player trials, when the participants made different choices, resulted in an amplification of the emotional responses, as also indexed by SCR (**Fig. 3B**). Thus the interdependence (based on social status) between the two participants strengthens the emotional experience when assessing the consequence of one's choice.

### Social gains loom larger than social losses

**Figure 4:**
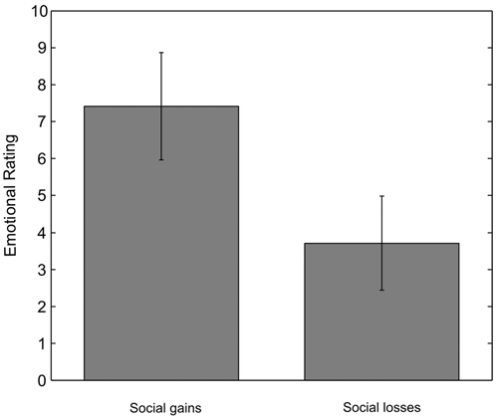
Difference between subjective evaluations in social and private domains. On the left we report the difference for gains (gloating minus relief), on the right the one for losses (regret minus envy).

The emotional evaluation was significantly higher (WSRT, *Z* = 1.989, *P* = 0.046) for social gains than for social losses (**Fig. 4**). Moreover, gloating is the emotional response with the highest SCR (**Table S1**). Thus, contrary to the private domain [3], [30], social gains loom larger than social losses. We performed an ANOVA on mean emotional ratings of private gain (of relief minus rejoice, M = −2.68, SEM = 2.22, relief and rejoice were not significantly different, *P* = 0.2), private loss (disappointment minus regret, M = 6.16, SEM = 3.26), social gain (M = 7.41, SEM = 1.45) and social loss (M = 3.71, SEM = 1.27). The ANOVA revealed a significant interaction between domain (private vs. social) and valence (gain vs. loss, F(1,40) = 6.949, *P* = 0.012). We conclude from this analysis that indeed in the private domain, losses loom larger than gains while in the social domain, gains loom larger than losses.

### How social emotions affect choice behavior

Subjective ratings, SCR and heart rate measurements might simply indicate affective responses with no consequence on behavior. Our model of choice with interdependent utilities (see **Methods** and **Supporting Text S1**) suggests otherwise: once participants experience the fact that the others' choice will affect the utility they derive from their outcome they will anticipate this effect on future trials, and take it into consideration at the moment of choice by accounting for anticipated emotions.

The experiment was designed to analyze this effect, by randomly allocating participants to two treatments that we may call bold and prudent. In the bold one, participants were facing the choices of the other as determined by a computer programmed to select the lottery with the higher expected value, irrespective of the risk. In the prudent one, the choices of the program minimized the variance of the outcomes of the lottery and therefore were those of an extremely risk averse decision maker. The use of two different criteria (expected value and risk) implied that the choice of the opponent that participants were facing differed on a substantial part of the trials (see **Methods**). In other words, the two groups of participants were facing two different competitors: one group had tough competitors, with high average payoff, the other weaker competitors with relatively lower average earnings.

The model we report (see **Methods** and **Supporting Text S1**) predicts that if participants derive more utility from social gains (gloating) than dis-utility from social losses (envy), their behavior in the two environments will be significantly different, and dependent on the behavior of the opponent. The dependence is not based on imitation (in line with [31]): Rather than adjusting to the different environment by mimicking the behavior of the other, participants should behave boldly in the prudent environment, and prudently in the bold one.

The evidence provided by subjective emotional evaluations and SCR data suggests that participants are indeed more sensitive to social gains than to social losses, so the condition that participants like winning more than they dislike losing is satisfied. Therefore we should observe that the behavior of participants is the opposite of that of their opponent.

### Effect of the environment on choice: evidence for complementary behavior

A simple way of measuring this effect is to estimate the contribution of the lotteries' expected value and standard deviation (risk) to the probability of choice. Regression analysis on choice behavior over all participants and trials show that the estimated coefficients for each of these two variables have the expected sign: a higher expected value increases the probability of choice of the lottery; a higher variance reduces this probability (**Table 1**). Moreover, the participants were risk averse in the gain domain and risk seeking for loss (**Table 1**: the variable giving the interaction between risk and loss is positive, *P* = 0.001), as predicted by Prospect Theory [32]. Notably, there was no difference in the way males and females made their choice in term of risk end expected value.

**Table 1:** Regression analysis of participants' choice.

Variable name	Coefficient	Standard error	Z	*P*
dev	0.276	0.012	22.42	<0.001
dsd	−0.031	0.010	−3.16	0.002
dsd*loss	0.048	0.015	3.24	0.001
constant	−0.121	0.041	−2.94	0.003

Number of subjects = 42; number of observations = 3360. Data from all trials (t = 80).

Log likelihood = −1944.3451, Wald chi2(3) = 596.32, Prob>chi2 = 0.000.

The table reports the coefficients estimated in the logistic regression of the choice made by participants. The dependent variable *choice* is equal to 1 if the participant chose the lottery 1 and 0 if the participant chose the lottery 2. The variable dev is the difference between the expected value of the first and second lottery (when participants maximize expected values the coefficient is positive); the variable dsd is the difference between the standard deviation of the first and second lottery (a negative coefficient indicates participants' risk averse behavior). The variable dsd ^*^ loss is equal to the latter when the expected value of the two lotteries is negative: this coefficient captures the loss aversion of participants.

**Figure 5:**
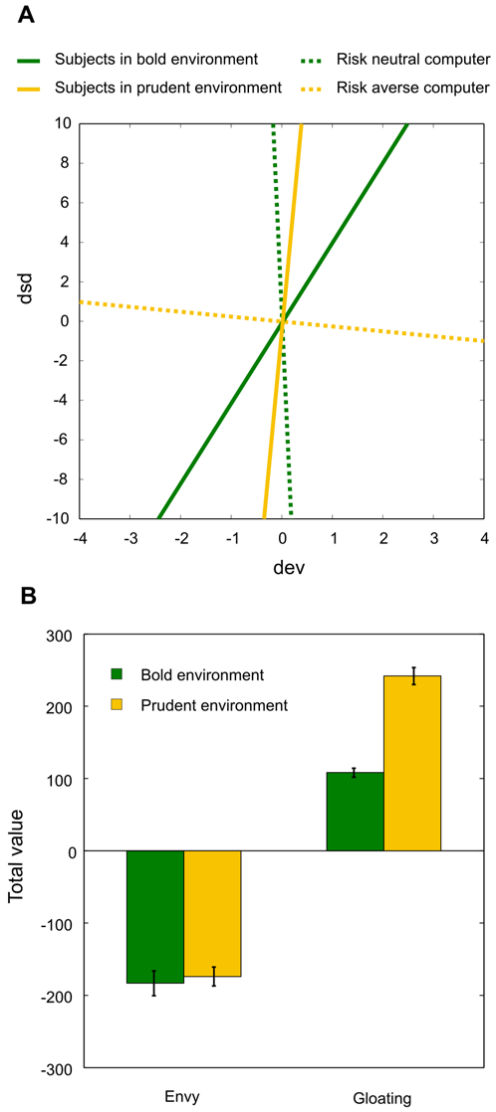
Effect of the environment on choice. (a) Participants behaved boldly in the prudent environment and prudently in the bold one. The lines describe the choice behavior of participants in the two environments (continuous green and yellow lines) and of the two computers (dotted lines). The figure is based on the logit analysis of the choices of participants and of the computers in later trials (t>40). The horizontal axis reports the difference between the expected value of the first and second lottery (dev), and the vertical axis the difference between the standard deviations (dsd). The lines have slope equal to minus the ratio of the coefficient of dev and the coefficient of dsd. A flatter slope corresponds to higher risk aversion. For example, a zero coefficient on the difference in standard deviation (the participant is indifferent to the risk of the lottery) produces a vertical line. A zero coefficient on the expected value (the participant is only sensitive to risk) produces a horizontal line. (b) The experience of gloating induced the behavioral change. The total experienced emotions in each environment averaged across participants. On a single trial we measured the difference between the obtained outcome and the outcome of the unchosen lottery. For each event we then summed these differences to compute the total value of each experienced emotion.

When considering the effect of the two environments, the results showed no difference in choices made in the initial trials in which the participants in the two groups observed the same choices of the opponent; but a significant difference appeared in later trials (**Tables S6** and **S7**). **Figure 5A** shows that in later trials participants in the bold treatment (risk neutral computer) became relatively more risk averse, while participants in the prudent treatment showed the opposite pattern of behavior.

### Experienced emotions, anticipated emotions, and choice

What produced this difference in choice behavior between the two groups? The participants in the two groups experienced very different relative payoffs compared to the opponent, and this induced different emotional experiences. These differences can be measured by the frequency of occurrence of the various emotional events, and by the average difference in the payments for the two participants in that event. For example, the measure for gloating is provided by the difference between the participant's payoff and the opponent's payoff.

The only type of event for which the difference is significant is gloating (when we consider number of occurrences or value), and this difference is large (**Figure 5B** and **Table S8**). Participants facing a prudent opponent had a proportion of trials in which gloating was experienced that was double the proportion of the same type of trials for participants facing a bold opponent (**Table S9**, 13.45 per cent instead of 7.08, that is a 6.37 per cent difference, Mann-Whitney *U* test: *Z* = 5.243; *P*<0.001). The average total dollar value of gloating was 133 dollars more for the participants in the prudent treatment than for those in the bold treatment (Mann-Whitney *U* test: *Z* = −5.137; *P*<0.001). The difference for envy is smaller and non-significant (Mann-Whitney *U* test: *P*>0.2): 10.3 instead of 9.1 for the frequency of occurrence, and 174 instead of 183 for the difference in value.

The cumulated effect of this difference over trials is likely to affect behavior: participants who experience gloating in the past may be more likely to make risky choices in the future. To test whether past gloating affects behavior, we computed the average value of the difference in payment associated with type of events in the first 40 trials, and tested the effect they had on choices made in the later trials. For example, the mean value of the envy is measured by the mean value of the difference between the opponent's payoff and the participant's payoff in the early envy trials. The past experience affects choice in the later trials: in complete agreement with the data provided by the subjective evaluations, gloating has a strong and significant effect, and reinforces risk loving behavior (*P* = 0.021 for the estimated coefficient in the panel logit regression: see **Table S10**); the marginal effect is 3.18 percentage points to the dollar.

The difference in choice behavior between the two groups of participants is the joint consequence of the effect of gloating on risk aversion and the difference in the amount of gloating experienced by the participants. Both are influenced by the past choices of the participant and of the other player. Gloating is the only emotion that shows a significant marginal effect (**Table S10**) as well as a large difference among the total amounts experienced by participants. The net effect is the significantly higher level of risk loving behavior in participants in the prudent treatment. In conclusion, the environment in this experiment influences behavior. The way in which this happens is not by imitation, but by producing the most rewarding behavior in a competitive environment.

## Discussion

The theoretical model that we present predicts that socially motivated emotions like envy and gloating combine the learning function (that they share with emotions like regret and relief) with the response to changes of one's social status.

We have three main findings. Emotions in the two-player condition, for the events in which participants made different choices, are stronger than in the single player condition. The second result is that social emotions operate differently from private ones: while regret looms larger than relief, gloating looms larger than envy. The third result is that participants behaved boldly in the prudent environment (against a weak opponent) and prudently in the bold one (against a bold opponent).

Our initial hypothesis was that socially motivated emotions like envy and gloating combine the learning function (that they share with emotions like regret and relief) with the response to changes of one's social status. Indeed, we saw that both components are relevant. Emotions in the two-player condition, when the participants made different choices, were stronger than in the single player condition.

The effect in not induced by any social emotion (as opposed to non-social) as shared regret and shared relief received weaker ratings than regret and relief experienced in a non-social context. Thus, envy and gloating matter more because they are socially competitive emotions, not just interpersonal ones. Moreover, this effect takes place even if the interaction between the two participants was minimal: they were clearly instructed that the payment would not depend in any way on the performance of the other participant. One possible hypothesis is that the effects we find are due to attention drawn to the lottery chosen by the other player. We tested this hypothesis by comparing the evaluations in single player and shared outcomes events, since the attention hypothesis suggests that there would be no difference between private and shared emotions: but this was not the case. Besides, even when both players made the same choice the payoff of the non chosen lottery influenced emotional ratings while the unobtained payoff of the chosen lottery did not (**Table S5 and Supporting Text S2**).

There are many factors influencing our status (that is, the position relative to others) following the observation of the outcome. One factor is the outcome itself (the payment of the lottery, in our experiment) that can for example affect our relative wealth position. This factor would operate even if a good outcome is completely due to chance, and does not signal anything on our ability. A second factor is the updating of the opinion we have of our own ability, we feel better if someone else does not do well, because our opinion on our current relative position is improved. Yet another factor is the observation by a third party of the relative performance. In this case too a poor performance of the others is better because our relative standing improves.

The theory we present predicts the economic analogue of the dominance complementarity observed in postural relationship [31], where a dominant posture is likely to induce a submissive one, and vice-versa. We observed in fact that participants behaved boldly in the prudent environment (against a weak opponent) and prudently in the bold one (against a competitive opponent). It is interesting to note that this effect seems to happen outside the participants' awareness. They reported in an ex-post debriefing questionnaire that they had not been influenced by the other player's choice behavior.

**Figure 6:**
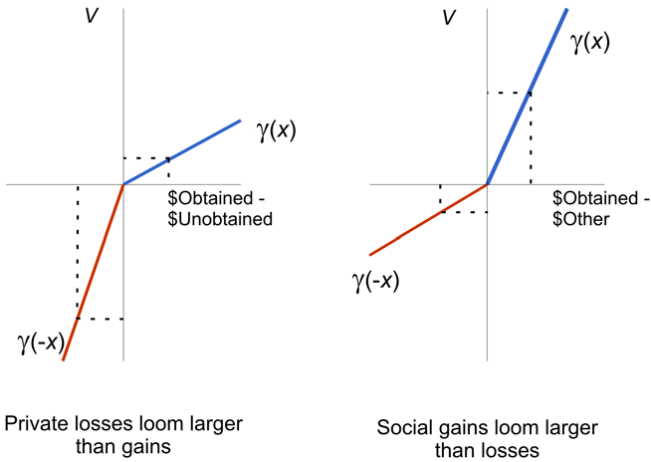
Social gains loom larger than social losses. The figure illustrates the relationship between the theory we suggest (IUT) and Prospect Theory [32]. The horizontal axis in both panels reports the difference between the outcome for the counterfactual choice and the outcome of the choice made by the agent. In the left panel the counterfactual choice is the choice that the agent could have made; in the right panel the counterfactual choice is the choice of the other agent. The vertical axis reports the utility derived from the comparison of obtained and counterfactual outcome. In private comparisons, losses have a larger effect on utility than gains. In the social comparison the opposite is true. The utility function reported in the left panel is similar to the value function for gains and losses assumed in Prospect Theory [32], where also losses loom larger than gains (loss aversion).

Social emotions operate differently from private ones: while regret (private loss) looms larger than relief (private gain)[2], [3], gloating (social gain) looms larger than envy (social loss) (**Fig. 6**). In the theory that is the background of the paper (IUT) choice is based on the anticipation of the emotion that the individual will experience after the outcome has been revealed. Figure 6, contrasting the value of private versus social gains and losses, is within this conceptual framework. It describes anticipated emotions. How are anticipated and actually experienced emotions linked? We provide insights on the interaction between the two. The data on choice allow us to estimate this anticipated state. The data on ex post evaluation allow us to measure experienced states. For example, the influence that past experiences have on later choices is produced by the actual experience of envy, gloating, and other emotions that is the way in which experienced emotions modify the anticipated emotions.

These findings suggest an important difference between the private and the social dimensions. In both cases deviations from expected utility are explained by the effect of the difference between the obtained outcome and the alternative possible outcome. In the private domain, the alternative outcome is that of an action that was not chosen, and aversion to loss (regret) dominates. This is similar to the loss aversion in Prospect Theory [32]. In the social environment the alternative outcome is that of a choice made by another person, and love of gain (gloating) dominates. Among animals, there are strong incentives for wanting to be at the top of the social ranking. Animals in the dominant position use their status to secure privileged access to resources, such as food [33] and mates [34].

To explain the difference between the relative weight of gains and losses in private and social environments one may consider the different impact of gains and losses in the private and social environments. In private environments, losses are particularly harmful because they can bring an individual closer to a critical level in terms of survival. Hence losses have to be avoided more than gains. In social environments, rewards are frequently assigned on the basis of a winner-takes-all rule: this is true for example in sexual competition. With this rule, being first is much better than being second, but the difference between second and third is not large, since with a winner-takes-all rule the outcome is the same for second and third. Hence behavior is driven more by the prospect of winning than the prospect of losing.

## Methods

### Participants

Forty two participants participated in the experiment (29 males). The average age was 21.5 years (±2.01 years). They were recruited via an online recruitment system. They were students at Lyon University, who had previously joined the recruitment system on a voluntary basis. These volunteers gave written informed consent for the project which was approved by the French National Ethical Committee.

### Experimental design

Each experimental session lasted 80 trials. Participants did not know each other before the experiment, and met at the beginning of the session. They sat in the same room, each playing on a computer, separated by a panel wall. They were told they were about to play together at the same game but that their own gain would not depend on the other's choices. In each trial participants chose one of two lotteries. A lottery is a description of two monetary outcomes, each with a probability indicated by a sector on a circle. All participants were presented with 40 lotteries in the single player game, and 40 in the two player game. The set of choices in the single and two player game were identical, and presented in an embedded way. The order of presentation was pseudo-randomized and was the same for all participants. The lotteries consisted entirely of combinations of four possible outcomes: −20,−5, 5 and 20. The probabilities of different outcomes were in the set {0.2, 0.5, 0.8}. During training the participants saw their opponent real choices. However choices of the second player were computer simulated during the task to control the experimental environment. In addition, this allowed us to consider each participant as an independent observation.

### Lotteries

The lotteries were paired so that no one of the two would exceed the other in the first order stochastic dominance. In all choices, the expected value of both lotteries is either positive (in 5 pairs of lotteries) or negative (in the other 5). In 4 out of 10 pairs of lotteries one of the lotteries had a higher expected value while the other one had a lower standard deviation. In the remaining pairs, one lottery had both a higher expected value and a lower standard deviation than the other one. Pairs of lotteries were presented twice in each of the four blocks, once in a one player trial, and once in a two player trial. The order of the trials was randomized within each block (see **Annex S1** for a list of all pairs of lotteries used in the experiment).

### Procedure and choice Task

The participants were instructed that they were about to play a game with another person, that both players were going to be presented with the same trials at the same time, and that they would sometimes be able to see the other player's choice. The written instructions referred to the other as “the other participant” or “the other player”, never as an opponent. It was also emphasized that their final earnings would not be dependent on those of the other. The participants knew at the beginning of the trial whether it was going to be a one player or a two player trial. Each lottery was surrounded with one dotted square in case of a one player trial or two dotted square of different color (yellow and green: each color standing for each of the players), in case of a two players trial. Participants could choose at any time after the beginning of the trial. The other player choice was displayed always after one's choice had been made. After choice, the participant observed an arrow spinning, and stop, on both lotteries. He would then know how much he had won and how much he would have won choosing the other lottery. In the two player condition, the participant would also discover how much the other player had won. At the end of each trial, participants had to evaluate their emotional state. At the end of the experiment, participants were provided with a complete oral debriefing explaining that they did not see each other's actual choices and the reasons for the use of this deception. They were paid an amount of money corresponding to the average payoff of ten randomly selected trials.

### Two computer algorithms

During training the participants saw their opponent real choices and believed this was also the case during the game. However choices of the second player were computer simulated during the task in order to control the experimental environment. In addition, this allowed us to consider each participant as an independent observation. No participants reported any doubt about who they were playing with during post-task debriefing. One computer was choosing the lottery with the highest expected value in 90 per cent of the choices, and the other the lottery with the lowest standard deviation. Thus, the choice of the opponent that participants were facing differed on a substantial part of the trials, 24 of the 40 two player trials (in which the participant observed the choice of the opponent). The differences occurred at equally distributed points during the session. The average payoff was $ 4.125 per trial for the bold treatment and $ 1.875 for the prudent treatment. Thus, we had two experimental groups: 21 participants received a bold treatment, interacting with a risk prone opponent; and 21 participants had a more prudent treatment, facing a risk averse opponent.

### Event classification

Events were classified as follows. If the trial was a single player then the event was classified as regret if the outcome of the non-chosen lottery was larger that that of the chosen one, and relief in the opposite case. If the trial was a two player one, and participants had made a different choice, then the event was classified as envy if the outcome of the participant's lottery was smaller that the outcome of the other's lottery, and gloating in the opposite case. If the trial was a two player trial, and the two players had made the same choice, then the event would be shared regret or shared relief.

### Electrophysiological recording

Electrodermal skin conductance responses and heart rate were recorded with a BIOPAC MP35 data acquisition unit (BIOPAC Systems, EU), with a 500 Hz sampling rate. Experimental sessions took place in a noiseless room with temperature set to 20°c. ***SCR recording***. Two Ag/AgCl electrodes were placed on the non-dominant hand, after cleaning with neutral soap. The tension applied between the two electrodes was 0∶5 V. We considered, responses occurring between 1 to 3 seconds after stimulus onset and a delay between valley to peak inferior to 5 seconds [28], [35]. We kept responses with amplitude greater than 0,02 µS [28]. Mean SCR magnitudes were used when averaging size of SCR across trials. Absence of measurable responses was treated as response with amplitude zero. ***Heart rate***. Two electrodes were placed on the chest. Heart rate was computed for the 3 seconds following the display of the outcome of the lotteries. The variation was then computed by subtracting the heart rate during 2 seconds before the outcome (spinning period) from the computed heart rate.

### Theoretical model

We consider the value *V* when the participant chooses the act (lottery) *f* and the alternative is *g* of the simple form:
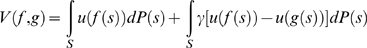
(1)Where *S* is the state set (i.e., all the possible outcomes), *P* the subjective probability on it, and *u* is the utility function. In the one-player trials the act *g* is the act that the participant has not chosen. The theory incorporates in its second component, described by the function γ, emotional responses to the difference (counterfactual) between the selected and the unselected act. In the more general model the function γ depends on the two terms separately, not simply on the difference of the utility of the two outcomes. So when the two outcomes are the same the value of this term is not zero. The functional form is the same, but the specific γ function is different in the single and in the two player environment. In the single player environment it only captures the counterfactual comparison of the obtained and unobtained outcome. In the two players it includes both this counterfactual evaluation and the emotional effect derived from social ranking. The crucial property of the function γ is the relative weight of gains (*u*(*f*(*s*))>*u* (*g*(*s*)) and losses (*u*(*g*(*s*))>*u* (*f*(*s*)). We can measure, as in Prospect Theory [32] the relative weight of gains and losses. Loss aversion in private choices can be formally described as the condition that, for any possible value (*x*) of the difference between the expected outcomes of the selected and the unselected act, −γ(−*x*)>γ(*x*), losses looming larger than gains. In the two-player trials, *g* is the act chosen by the other participant. If social losses loom larger than gains, −γ(−*x*)>γ(*x*) (envy dominates gloating) equilibria are symmetric, and the model (*Theory of interdependent utilities*, see **Supporting Text S1**) predicts same behavior for the two participants; instead if gains loom larger than losses, γ(*x*)>−γ(−*x*), (gloating dominates envy) the equilibria are asymmetric, and the behavior of participants should be the opposite of that of their opponent, seeking for differences in final incomes.

### Statistical Analysis

The analysis was conducted with the statistical software package Stata, Stata Corp, College Station, TX, Release 9/SE. Non-parametric tests were applied on the data sets since it violated several parametric assumptions, particularly non-normal distribution of the data and high proportion of zero responses (in case of SCR magnitude). We found no evidence of habituation effects across the experimental session. The significance of the difference between behavioral variables, response time and subjective evaluations is estimated with the Wilcoxon signed rank test (non parametric test, [5]); the hypothesis tested is that the distribution of two random variables for matched pairs is the same. A Bonferroni correction is used in order to correct for multiple comparisons. Between groups differences were tested with Mann-Whitney U test. Choice behavior was analyzed based on panel data analysis, which takes each participant as the unit and the round as time. Both random and conditional fixed effects were estimated, and we report the results for the random effects analysis. The parameters are estimated by maximum likelihood.

### Choice behavior

The model used to estimate the parameters of the choice of players is the logit:
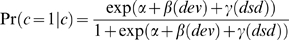
(2)where if *ev*_i_ is the expected value of lottery i; i = 1; 2, then *dev*≡*ev*_1_−*ev*_2_. Similarly, if *stdev*_i_ is the standard deviation of the value of lottery i; i = 1; 2, then *dsd*≡*stdev*_1_−*stdev*_2_.

### Measuring the effect of the environment on choice

We estimate with the logit regression (**Tables S6** and **S7**) the probability of the participant choosing the first lottery, as a function of the difference in expected value and standard deviation. The behavior of participants in the prudent environment is risk neutral: the *dsd* coefficient is very small (in absolute value). Risk significantly predicts choices of participants in the bold environment. The *dsd* coefficient is negative, which means individuals minimize the risk when choosing; then this group is risk averse. In **figure 5a**, we estimate with the logit regression the probability of choice for the two groups (**Tables S6** and **S7**) and the two computers. The lines describe the choice behavior of participants in the two environments (continuous green and yellow lines) and of the two computers (dotted lines), as follows. We obtain the three coefficients α, β and γ from the regressions for each group. One (α) would indicate a bias between the lottery 1 and lottery 2 and in fact is not significantly different from zero. The coefficient β for the difference in expected value is positive. The last one, γ for the difference in the standard deviation is negative. The two latter coefficients give a measure of the tradeoff between the two variables *dev* and *dsd*. The lines are the lines passing through the origin and with slope equal to minus the ratio of the coefficient of the expected value and the coefficient of the standard deviation, which is 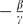. A flatter slope corresponds to higher risk aversion. For example, a zero coefficient on the difference in standard deviation (the participant is indifferent to the risk of the lottery) produces a vertical line. A zero coefficient on the expected value (the participant is only sensitive to risk) produces a horizontal line. The lines can be interpreted as the combination of difference in value and difference in standard deviation that give constant probability of choosing the first over the second lottery; as they pass through zero, this constant probability is 1/2, the probability of choosing lottery 1 over lottery 2 when they have same expected value and same standard deviation. Horizontal translations of these curves give constant probability: as they move to the right, the probability of choosing the first lottery increases.

## Supporting Information

Text S1 Interdependent Utilities Theory
(0.06 MB PDF)

Text S2 Alternative interpretation: Discussion of the effect of attention
(0.01 MB PDF)

Table S1 Subjective ratings, skin conductance responses (SCR), and heart rate variations for the different emotions. The magnitude of the SCR is computed for the moment in which the outcomes of the two lotteries are displayed (N = 42).
(0.06 MB TIF)

Table S2 Wilcoxon signed-rank test on emotional ratings for negative emotions. The null hypothesis is that the two ratings are the same (N = 42).
(0.07 MB TIF)

Table S3 Wilcoxon signed-rank test on emotional ratings for positive emotions. The null hypothesis is that the two ratings are the same (N = 42).
(0.07 MB TIF)

Table S4 Effect of obtained and unobtained payoffs on subjective ratings. A test of the effect of unobtained outcomes on the emotional ratings is provided by the regression. The regression shows that even if the unobtained outcome of the chosen lottery has an effect on the emotional ratings, it influences significantly less the ratings than the outcome of the non chosen lottery (Chi^2^ = 7.71, p = 0.0055). Moreover, the regression coefficient of the TPD (two players, different choice) dummy is positive and significant in this regression. Thus, the amplification in evaluations due to envy and gloating, in the two player trials when the two players made different choices, is still significant when taking into account the potential effect of the unobtained outcome of the chosen gamble (i.e., disappointment and elation).
(0.10 MB TIF)

Table S5 Effect of obtained and unobtained payoffs on subjective ratings in the two player condition with same choice.
(0.07 MB TIF)

Table S6 Choice behavior in the prudent environment. The table report the coefficients estimated in the logistic regression of the choice made by participants in the prudent environment, in the two player condition for late trials (trials>40). The dependent variable choice is equal to 1 if the subject chose the lottery 1 and 0 if the subject chose the lottery 2. The variable dev is the difference between the expected value of the first and second lottery (when participants maximize expected values the coefficient is positive); the variable dsd is the difference between the standard deviation of the first and second lottery (a negative coefficient indicates participants' risk averse behavior). The behavior of subjects in the prudent environment is risk neutral: the dsd coefficient is very small (in absolute value).
(0.17 MB TIF)

Table S7 Choice behavior in the bold environment. Same estimate as in table S6, for participants in the bold environment, in the two player condition for late trials (trials>40). Risk significantly predicts choices of subjects in the bold environment. The dsd coefficient is negative, which means individuals minimize the risk when choosing; then this group is risk averse.
(0.17 MB TIF)

Table S8 Experienced emotions. The total experienced emotions in each environment averaged across subjects. On a single trial we measured the difference between the obtained outcome and the outcome of the unchosen lottery (in absolute value). For each event we then summed these differences to compute the total value of each experienced emotion. For instance, the total value of gloating is defined as the sum of the differences between the outcome of the lottery chosen by the other subject and your outcome, when this difference is unfavorable.
(0.06 MB TIF)

Table S9 Average over subjects of the number of occurrences of each event in both environments.
(0.06 MB TIF)

Table S10 The effect of experienced emotions on choice. The table reports the coefficients estimated for the average value of the difference in payment associated with different events in the first 40 trials (early envy and early gloating respectively) on choices made in the last 40 trials. The variables dev, dsd and dsd *loss are as in Table 1. The two last variables are the product of the total value of envy and gloating in the early (first 40) trials times the variable dsd.
(0.10 MB TIF)

Annex S1 Pairs of lotteries used in the experiment
(0.12 MB TIF)
