# Supplementary material for: Interdependent Utilities: How Social Ranking Affects Choice Behavior
Source: PLoS One. 2008 Oct 22;3(10):e3477. doi: 10.1371/journal.pone.0003477 (PMC2568945; doi:10.1371/journal.pone.0003477)
Supplement: Text S1 — Interdependent Utilities Theory (0.06 MB PDF) [file pone.0003477.s001.pdf]

Interdependent utilities: How social ranking affects choice behavior

Supporting Information

The relationship between regret and envy is intuitively clear: in both cases, a subject compares the outcome that he has received from the choice he made with what he could have received with a different choice. The only difference between the two emotions is that in the case of regret the different choice is made hypothetically by him, and in the case of envy by someone else. In both cases the crucial element is the counterfactual thought of what could have happened had the subject made a different choice.

We present here a model that makes this intuitive notion precise, and can be used in the statistical estimates of our data. The model is based on the classical setup of Anscombe and Aumann [1], and is developed in detail in Maccheroni, Marinacci and Rustichini ([2]), elaborating on methods in [3], where a precise axiomatic foundation for the utility is presented. The model provides sound behavioral foundation for choice where the utility depends on utilities.

1 Choices and consequences

There is a set of states S and a set C of consequences. An act is a function f from the set of states to the set of consequences. For any given act f, the realization of a state s determines the consequence $f(s)$ delivered to the subject. A set of acts F is available to the subject, when he chooses in isolation, or to all subjects when they are informed about the choice and the outcome of others. Each subject has preferences, indicated by $\succeq$, over vectors $(f, (f_i)_{i \in I})$ of the chosen act f and the set $(f_i)_{i \in I}$ of alternative acts that were available to the subject, in the single player environment, or to all the players in the many players environment. The value of the pair for the subject keeps into account both the direct utility from the choice, as well as the relative comparison that will be made, for every state that realizes, between what the subject gets (defined by $f(s)$), and the consequences $(f_i(s))_{i \in I}$ obtained through the alternative acts.

2 Representation of preferences

The preference $\succeq$ satisfies a specific set of axioms (see [2] for details) if and only if there is a utility function u defined on C, a subjective probability P over S, and a function γ such that $(f, (f_i)_{i \in I}) \succeq (g, (g_i)_{i \in I})$ if and only if $V(f, (f_i)_{i \in I}) \geq V(g, (g_i)_{i \in I})$, where

$$V(f, (f_i)_{i \in I})) = \int_S u(f(s)) dP(s) + \int_S \gamma(u(f(s)), u(f_i)_{i \in I})) dP(s)$$

The function V is the sum of two terms. The first is the standard expected utility of the act f: An individual with no regret or envy (that is, with $\gamma = 0$) is an expected

utility maximizer. The second term is the expected value of a term which measures the utility (or dis-utility) deriving from the comparison between the realized utility and the utility associated to the relevant alternatives. For both terms the expectation is taken with respect to a subjective probability distribution over the states, indicated by P.

The function γ is increasing in the first term and decreasing in the second (if we give to the vectors with $\#I$ coordinates the point-wise partial order). The second term of this function summarizes the effect of the counterfactual thinking: if a higher utility is associated with alternatives that were not chosen, then the current utility decreases. When the subject is choosing in isolation, the comparison is made between the act he chose and the acts he could have chosen. When he is choosing together with other individuals, the comparison is between the act he chose and the set of choices made by the others. His value is increasing in his utility (that appears in both terms), and decreasing in the utility of the others.

3 Preferences and Choices

In our experiment, the set of choices has two elements, denoted by $\{f, g\}$, both in the case of the single player trials and in the two players trials. We consider the value V when the subject chooses f and the alternative is g of the simple form:

$$V((f,g)) = \int_S u(f(s))dP(s) + \int_S \gamma[u(f(s)) - u(g(s))]dP(s) \tag{3.1}$$

In the one-player trials the act g is the act that the subject has not chosen. In the two-player trial, it is the act chosen by the other subject. The function γ is increasing, and $\gamma(0) = 0$. The crucial property of this function is the relative weight of gains and losses.

4 Private *vs.* social gains and losses

An important component of Prospect Theory [4] can be informally described as "losses loom larger than gains". This condition can be more precisely stated either as a local condition at the reference point zero that the derivative from the left is larger than the one from the right. More globally, the condition can be states as $v(x) < -v(-x)$ for every x.

In the case of a subject choosing in isolation, this condition has a correspondent: the preference exhibits more regret than relief. Several studies of regret suggest that indeed in this case too the negative dominates the positive, and regret looms larger than relief. The evidence we have seen suggests that for social gains and losses, the gain may loom larger than losses. On the basis of the setup we have presented we can extend the analysis of the relative importance of positive and negative emotions to regret and envy.

If x is a consequence, we denote by x also the constant act giving the consequence x in every state. For an event A, and another consequence y, we denote by xAy the act that gives x on A and y in its complement. Consider for simplicity the case in which there is a single other player: $I = \{1\}$.

We say that a preference $\succeq$ exhibits more gloating than envy if $(x_o, xEy) \succeq (x_o, x_o)$ for every event E which has a subjective probability of 50 per cent, and such that the player is indifferent, in isolation, between xEy and x_o. The subject prefers the fair chance of winning some and losing some rather than tying all the times. It is proved in [2] that a

preference $\succeq$ exhibits more gloating than envy if and only if the function γ satisfies:

$$\text{for all } x,\ \gamma(x) + \gamma(-x) \geq 0 \tag{4.2}$$

For the case of regret, the formal statement is identical to that given above comparing gloat and envy, with the only difference that the act xEy is the act that the subject could have chosen but did not choose.

We now see what this property of the function γ implies on the behavior of players in social environments, like our two-player treatment.

5 The Nash equilibrium set

In the two players trials the subject has to keep into account, at the moment of selecting his lottery, the likely choice of the other player, because his value will be influenced by that choice as well as his own. The appropriate concept in this case is the Nash equilibrium of the game where each subject chooses an act out of the set $\{f, g\}$, and each player receives a payoff given by the function (3.1). The analysis of the equilibria of these game requires that we allow subjects to choose a probability distribution over acts (that is, a mixed strategy). We now analyze the structure of the Nash equilibrium set for this game.

The game is symmetric: if we denote the strategy profile $h = (h^1, h^2) \in \{f, g\}^2$ chosen by the two players, then the pair of payoffs to the two players is the pair:

$$(V(h^1, h^2), V(h^2, h^1))$$

Let p and q denote the probability that the subject and the opponent, respectively, choose the act f in the mixed strategy. The best reply of player 1 to the strategy q of player 2 is denoted $BR^1(q)$, a subset of $[0, 1]$. Since the game is symmetric, $BR^1 = BR^2 \equiv BR$. To characterize the structure of the Nash equilibria we only need to consider the best response to the extreme values of p and q, that is the values $BR(0)$ and $BR(1)$. The graph of the best response function will determine the set of equilibria. For example, if both values are $\{1\}$, then the only equilibrium outcome is (f, f); if $BR(0) = \{0\}$ and $BR(1) = \{1\}$ then there are three symmetric equilibrium outcomes, with outcomes (f, f), (g, g) and a mixed strategy equilibrium.

It is clear that $1 \in BR(0)$ if and only if $V(f, g) \geq V(g, g)$: that is, the choice of f (that is, $p = 1$) is optimal when the other player is choosing g ($q = 0$) if and only if f is at least as good as g when the other is choosing g. Similarly, $1 \in BR(1)$ if and only if $V(f, f) \geq V(g, f)$. The structure of the equilibrium set is completely determined by whether $V(f, g) \geq V(g, g)$ holds or not, and by whether $V(g, f) \geq V(f, f)$ holds or not.

The game may have symmetric equilibria in pure strategies with outcomes (f, f), (g, g) or both, depending on the function γ and on the two acts. For example, $V(f, g) \geq V(g, g)$ and $V(f, f) \geq V(g, g)$ if and only if (f, f) is an equilibrium outcome. The game has a non-symmetric equilibrium in pure strategies if and only if

$$V(f, g) \geq V(g, g) \text{ and } V(g, f) \geq V(f, f) \tag{5.3}$$

An equilibrium in mixed strategies (symmetric) exists if and only if two equilibria in pure strategies exist. Clearly, if $\gamma = 0$ then the only equilibrium is the one where both players choose the same act, the one that maximizes their expected utility.

6 Asymmetric Equilibria

Our experimental results suggest that the behavior of our subjects adjusts to an asymmetric equilibrium. In this equilibrium, one of the two players (the computer) is programmed in one treatment to select a safer choice, but with lower return, and in the other treatment to select a more risky but higher return choice. We have seen that the other subject adjusts to the behavior of the opponent by adopting an opposite behavior. In general, the existence of asymmetric equilibria implies restrictions on the function γ, that is on the way subjects' utility varies with the return of the other player. Clearly if such equilibria exist then the function γ is not zero: subjects must care about the outcome of the other. We know more: subjects must enjoy winning more than they dislike losing, at least for some values of the utility. To see this, suppose instead that:

$$\text{for all } x,\ \gamma(x) + \gamma(-x) \leq 0 \tag{6.4}$$

With this function the disliking of losing is larger in absolute value than the liking of winning. In this case if $V(f, g) > V(g, g)$ implies that $V(f, f) > V(g, f)$, and then an asymmetric equilibrium does not exist, since the condition (5.3) is not satisfied.

Functions γ for which the condition (6.4) does not hold, so that asymmetric equilibria are possible, are easy to find. A natural example is $\gamma(x) = x^{+}$: the subject only cares about the case in which he is first, and the utility is proportional to the size of the gap between him and the other.

The intuitive reason for this effect on behavior becomes clear if we consider the extreme case of two players with a risk-averse utility function who only care about winning. Suppose that the choice they have is between a certain reward of \$5 and a lottery offering amount of \$10 or \$0 with equal probability. If subjects are risk averse, they would choose the certain amount of \$5 in the one player condition. In the two player condition, a subject, lets call him Player 1, who knows that the other is going to choose the certain amount now has an additional incentive to choose the lottery: the gloating he enjoys when \$10 is the outcome. Suppose that this additional incentive is large enough to make him choose the lottery. Lets now consider the best course of action of the other player (Player 2): he could choose the lottery; but since the outcome is the same for both players, the total value he derives from it is equal to the expected utility of the lottery, which by risk aversion is smaller than the utility of the certain amount. In addition the choice of the certain amount gives the additional value from gloating when Player 1 gets the \$0 amount. Hence Player 2s best choice is the certain amount, and the Nash equilibrium is asymmetric.

References

[1] Anscombe, F. and Aumann, R. J., (1963): "A Definition of Subjective Probability," *Annals of Mathematical Statistics*, 34, 199-205.

[2] Maccheroni, F., Marinacci, M., and Rustichini, A., (2006), "Social Decision Theory". Mimeo.

[3] Maccheroni, F., Marinacci, M., and Rustichini, A., (2006), "Ambiguity Aversion, Robustness, and the Variational Representation of Preferences", *Econometrica*, November, 1-62.

[4] Daniel Kahneman and Amos Tversky, (1979), "Prospect Theory: An Analysis of Decision under Risk", *Econometrica*, 47, 2, 263-292.
