# Supplementary material for: Interdependent Utilities: How Social Ranking Affects Choice Behavior
Source: PLoS One. 2008 Oct 22;3(10):e3477. doi: 10.1371/journal.pone.0003477 (PMC2568945; doi:10.1371/journal.pone.0003477)
Supplement: Text S2 — Alternative interpretation: Discussion of the effect of attention (0.01 MB PDF) [file pone.0003477.s002.pdf]

Alternative interpretation: Discussion of the effect of attention

Supporting Information

The amplification of the emotional responses for two players trials, when subjects made differents choice (envy and gloating events) might also be interpreted as an effect of attention drawn toward the unchosen lottery, i.e. the lottery chosen by the other player. The attention of the participant might be directed to clues that are provided to him and influence the emotional ratings. In the present study, there are three conditions that may have different implications for attention: single player, (SP), Two Players Different Choice (TPD) and Two Players Same Choice (TPS). In TPS the lottery chosen by the two is highlighted, hence salient, while the other is not. So the attention model predicts that the attention is focused on the outcome of the chosen lottery, away from the non-chosen one. The same occurs in the SP condition; hence TPS and SP conditions are similar in term of attention. In the TPD, both lotteries are highlighted, hence salient. Hence the attention is drawn to both. Hence both outcomes are relevant. A first important prediction of the attention model is that TPS and SP should be identical. But they are not since the TPS emotional scores and GSR are smaller in size than in the SP. Another prediction is that the outcomes of the non chosen lottery in the TPS should not influence emotional ratings, since attention is drawn away form the non-chosen lottery. Table S5 shows the relative effects of the obtained and unobtained payoffs on emotional ratings in the TPS conditions: the outcome of the non-chosen lottery significantly affects emotional ratings while the unobtained payoff the chosen lottery does not. Thus emotional ratings are dominated by the comparison between the outcome of both lotteries, even when the two players made the same choice.

We can reasonnably conclude from these two findings that the results on emotional evaluation of the outcome cannot be solely explained by attention.
