# Supplementary figures and images for: Interdependent Utilities: How Social Ranking Affects Choice Behavior
Source: PLoS One. 2008 Oct 22;3(10):e3477. doi: 10.1371/journal.pone.0003477 (PMC2568945; doi:10.1371/journal.pone.0003477)

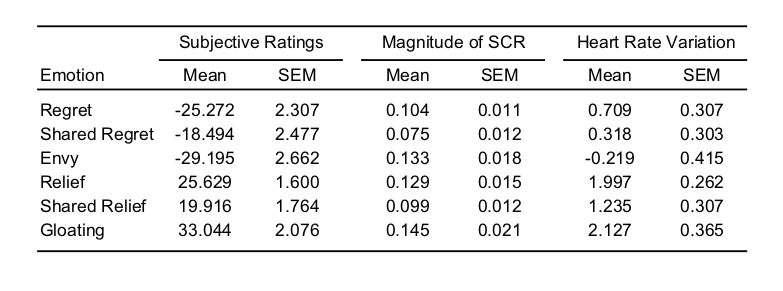

Supplement: Table S1 — Subjective ratings, skin conductance responses (SCR), and heart rate variations for the different emotions. The magnitude of the SCR is computed for the moment in which the outcomes of the two lotteries are displayed (N = 42). (0.06 MB TIF) [file pone.0003477.s003.tif]

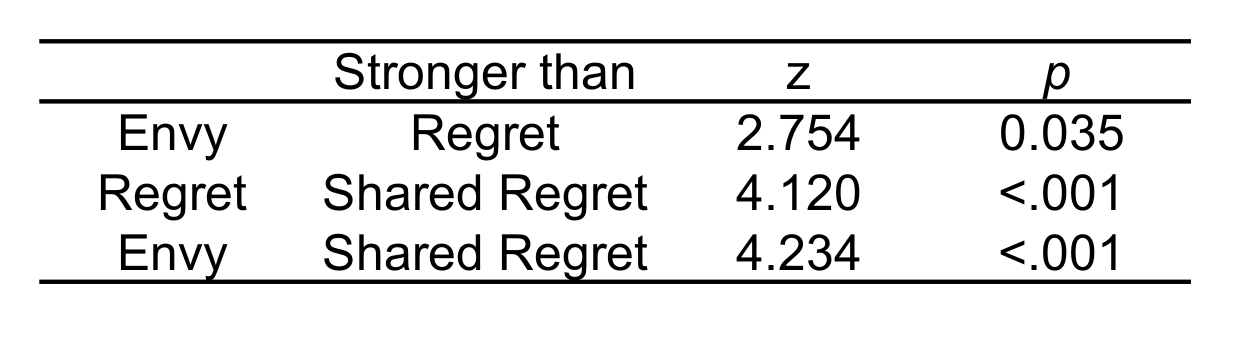

Supplement: Table S2 — Wilcoxon signed-rank test on emotional ratings for negative emotions. The null hypothesis is that the two ratings are the same (N = 42). (0.07 MB TIF) [file pone.0003477.s004.tif]

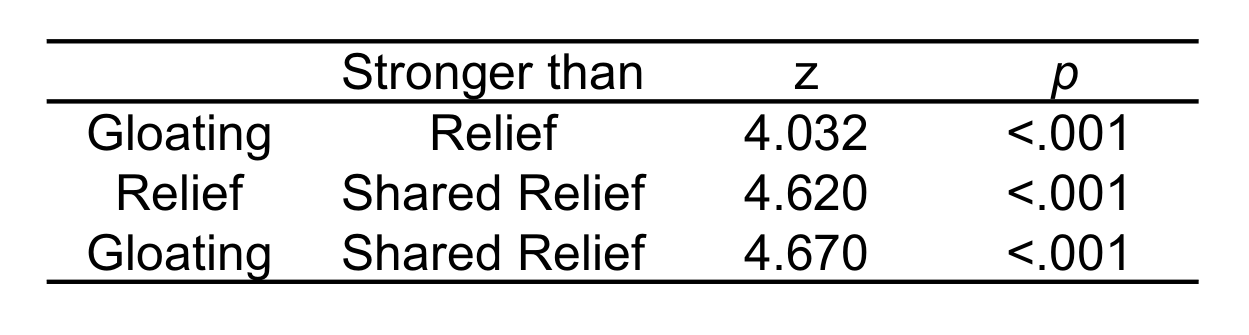

Supplement: Table S3 — Wilcoxon signed-rank test on emotional ratings for positive emotions. The null hypothesis is that the two ratings are the same (N = 42). (0.07 MB TIF) [file pone.0003477.s005.tif]

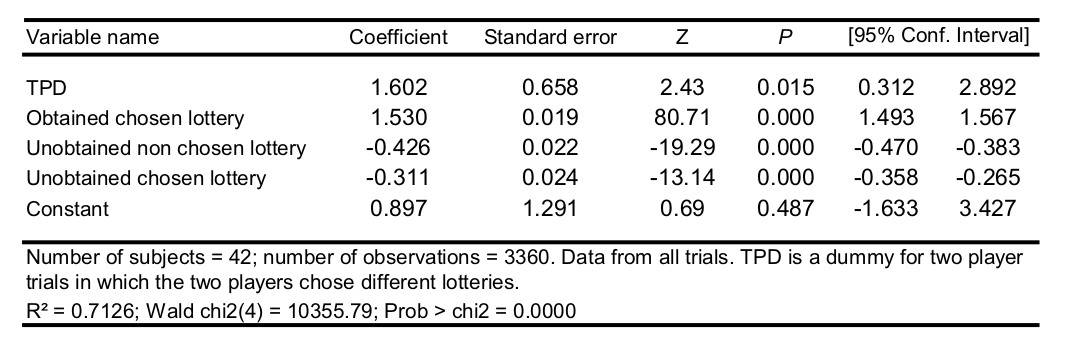

Supplement: Table S4 — Effect of obtained and unobtained payoffs on subjective ratings. A test of the effect of unobtained outcomes on the emotional ratings is provided by the regression. The regression shows that even if the unobtained outcome of the chosen lottery has an effect on the emotional ratings, it influences significantly less the ratings than the outcome of the non chosen lottery (Chi2 = 7.71, p = 0.0055). Moreover, the regression coefficient of the TPD (two players, different choice) dummy is positive and significant in this regression. Thus, the amplification in evaluations due to envy and gloating, in the two player trials when the two players made different choices, is still significant when taking into account the potential effect of the unobtained outcome of the chosen gamble (i.e., disappointment and elation). (0.10 MB TIF) [file pone.0003477.s006.tif]

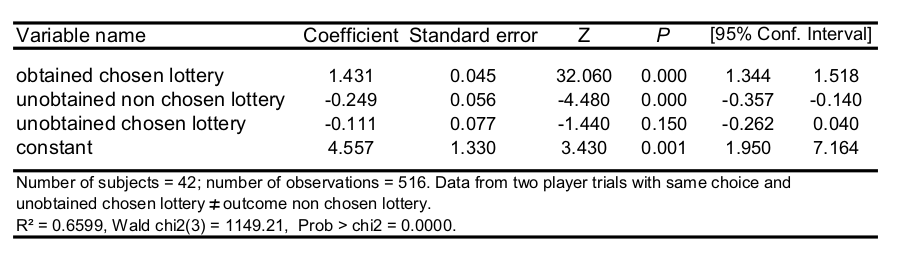

Supplement: Table S5 — Effect of obtained and unobtained payoffs on subjective ratings in the two player condition with same choice. (0.07 MB TIF) [file pone.0003477.s007.tif]

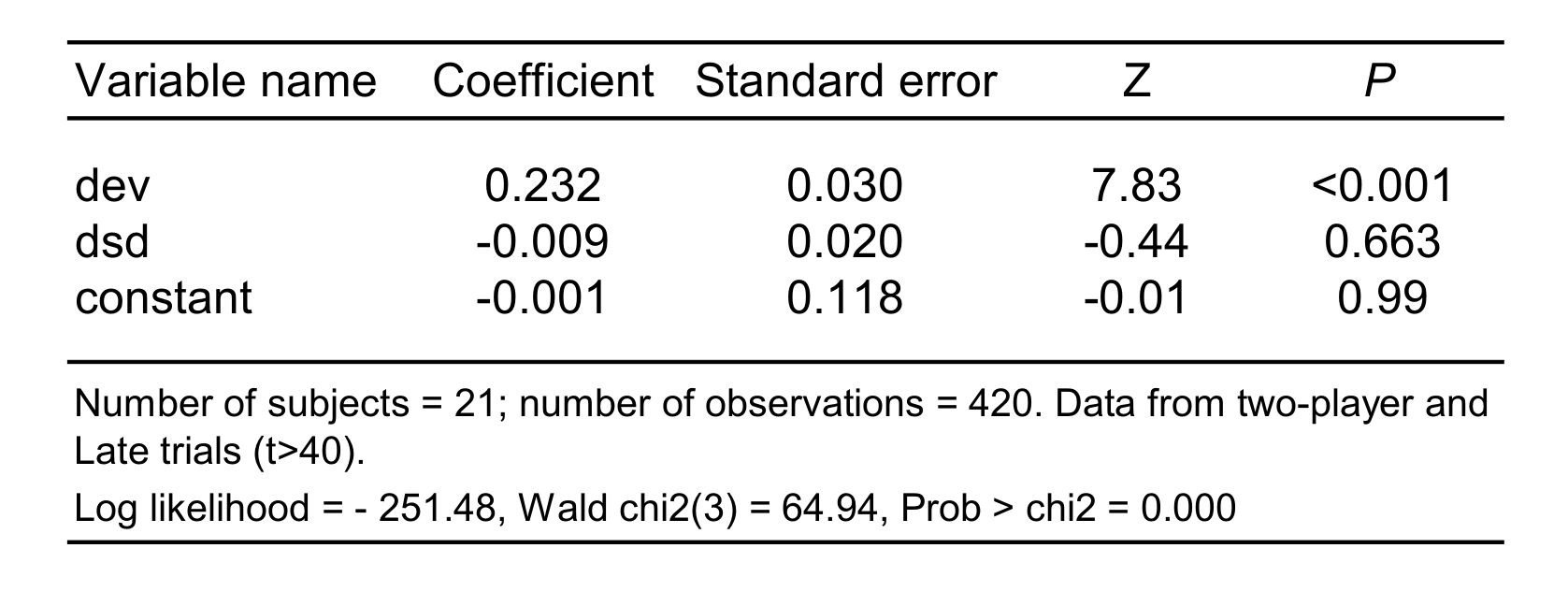

Supplement: Table S6 — Choice behavior in the prudent environment. The table report the coefficients estimated in the logistic regression of the choice made by participants in the prudent environment, in the two player condition for late trials (trials>40). The dependent variable choice is equal to 1 if the subject chose the lottery 1 and 0 if the subject chose the lottery 2. The variable dev is the difference between the expected value of the first and second lottery (when participants maximize expected values the coefficient is positive); the variable dsd is the difference between the standard deviation of the first and second lottery (a negative coefficient indicates participants' risk averse behavior). The behavior of subjects in the prudent environment is risk neutral: the dsd coefficient is very small (in absolute value). (0.17 MB TIF) [file pone.0003477.s008.tif]

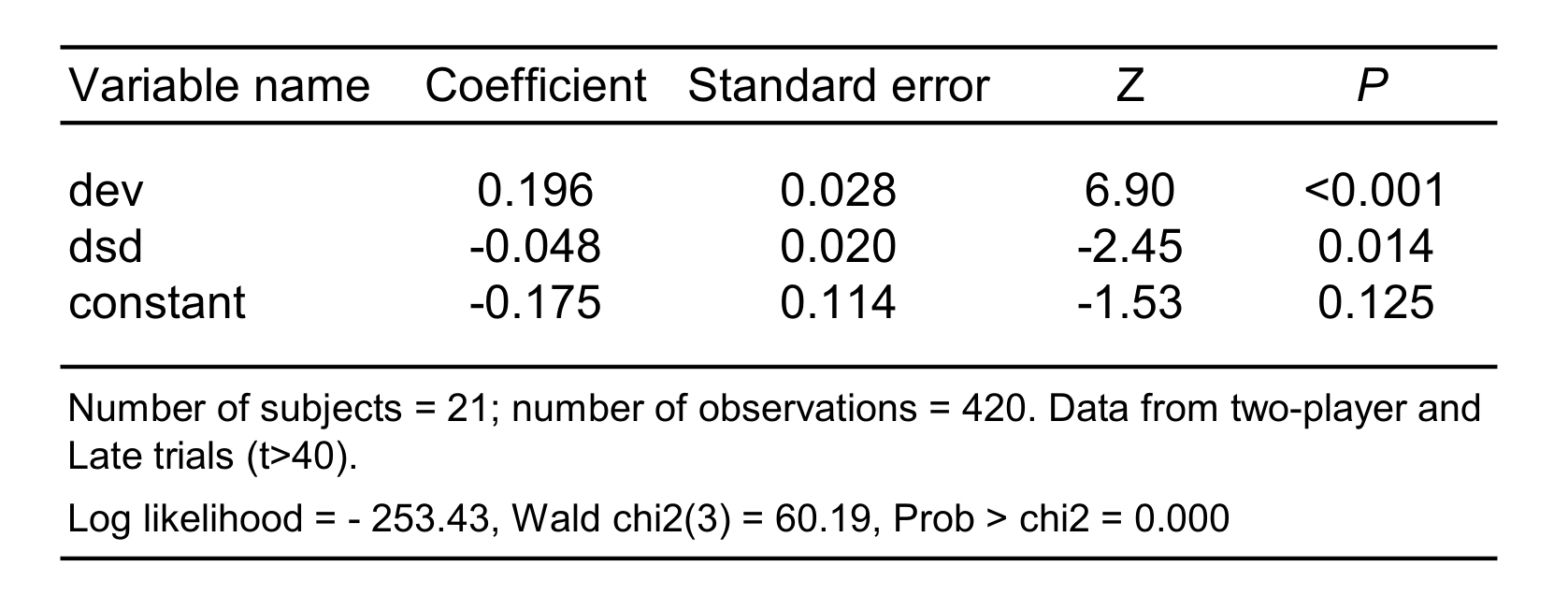

Supplement: Table S7 — Choice behavior in the bold environment. Same estimate as in table S6, for participants in the bold environment, in the two player condition for late trials (trials>40). Risk significantly predicts choices of subjects in the bold environment. The dsd coefficient is negative, which means individuals minimize the risk when choosing; then this group is risk averse. (0.17 MB TIF) [file pone.0003477.s009.tif]

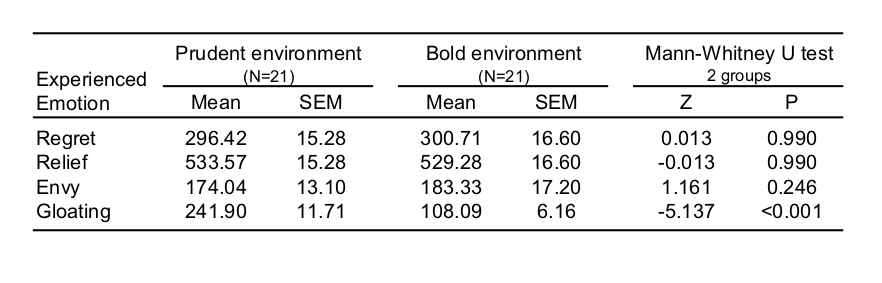

Supplement: Table S8 — Experienced emotions. The total experienced emotions in each environment averaged across subjects. On a single trial we measured the difference between the obtained outcome and the outcome of the unchosen lottery (in absolute value). For each event we then summed these differences to compute the total value of each experienced emotion. For instance, the total value of gloating is defined as the sum of the differences between the outcome of the lottery chosen by the other subject and your outcome, when this difference is unfavorable. (0.06 MB TIF) [file pone.0003477.s010.tif]

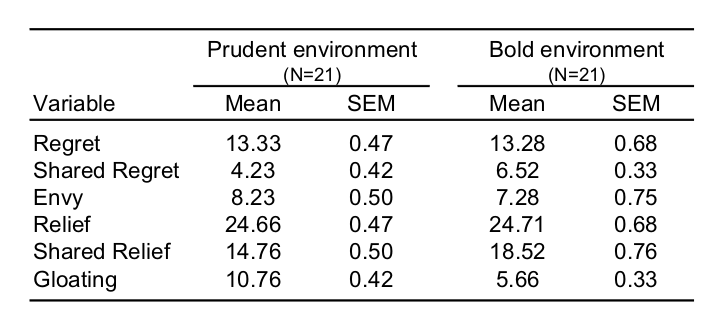

Supplement: Table S9 — Average over subjects of the number of occurrences of each event in both environments. (0.06 MB TIF) [file pone.0003477.s011.tif]

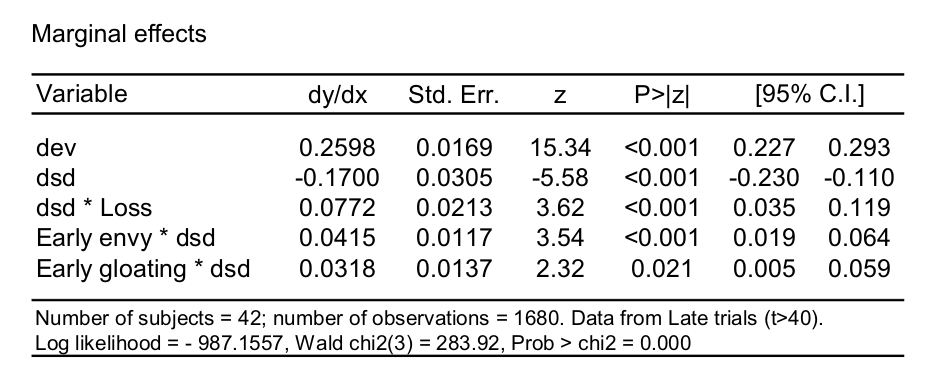

Supplement: Table S10 — The effect of experienced emotions on choice. The table reports the coefficients estimated for the average value of the difference in payment associated with different events in the first 40 trials (early envy and early gloating respectively) on choices made in the last 40 trials. The variables dev, dsd and dsd *loss are as in Table 1. The two last variables are the product of the total value of envy and gloating in the early (first 40) trials times the variable dsd. (0.10 MB TIF) [file pone.0003477.s012.tif]

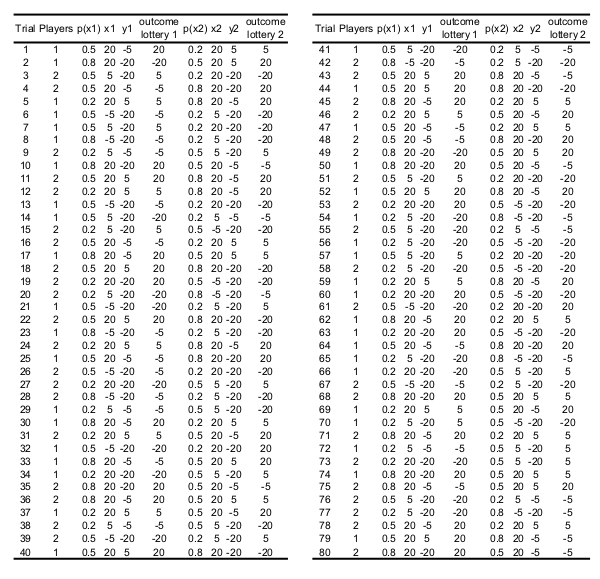

Supplement: Annex S1 — Pairs of lotteries used in the experiment (0.12 MB TIF) [file pone.0003477.s013.tif]
